# Chloronychia: Green Nail Syndrome Caused by Pseudomonas aeruginosa in a Younger Patient

**DOI:** 10.7759/cureus.83382

**Published:** 2025-05-03

**Authors:** Woodly T Dominique, Ebere Obasi, Shubman Sidhu

**Affiliations:** 1 Internal Medicine, Valley Health System, Las Vegas, USA; 2 Family Medicine, Memorial Health, Savannah, USA

**Keywords:** chloronychia, green nail, onycholysis, pseudomonas aeruginosa, younger age

## Abstract

This case report discusses a rare occurrence of chloronychia, or green nail syndrome, in a young woman in her 20s with a recent *Pseudomonas aeruginosa*-induced ear infection. Green nail syndrome is characterized by green nail plate discoloration, proximal paronychia, and distal onycholysis. The patient's infection likely originated from her recent ear infections and resulted in green discoloration on her right index fingernail. Her medical history revealed a complex ear infection treated with ciprofloxacin, clindamycin, and morphine. However, after the resolution of her ear infection, the patient presented with a prominent green discoloration in her finger with mild inflammation. She was treated with ciprofloxacin topical solution and bleach soaks, which led to the resolution of the symptoms. *P. aeruginosa*, an opportunistic bacterium, uncommonly causes nail infections, though rarely documented in younger patients. In this case, it spread from the ear to the fingernail, emphasizing the bacterium's opportunistic nature.

## Introduction

Green nail syndrome, also known as chloronychia, is characterized by proximal chronic non-tender paronychia, onycholysis, and green staining of the nail plate [1]. Chloronychia has been estimated to account for approximately 1-4% of all nail infections, with a higher prevalence in occupational settings where hands are frequently exposed to water [2,3]. The condition primarily affects adults between 40 and 60 years of age, with rare documented cases in patients under 30 years old [3].

*Pseudomonas aeruginosa* infection of the nail plate occurs in people whose hands are regularly exposed to water, soaps, and detergents or who are subjected to mechanical damage such as microtrauma, abrasions, or persistent pressure [2]. The characteristic green or black nail discoloration results from bacterial pigments pyoverdine and pyocyanin produced by *P. aeruginosa* [3]. Green or black nails should highlight the possibility of *P. aeruginosa* infection, which is typically treated with an oral quinolone (ciprofloxacin) [2].

This case report describes a rare occurrence of chloronychia in a young woman patient with a recent history of *P. aeruginosa*-induced ear infection, highlighting the opportunistic nature of this pathogen and its potential for cross-site infection.

## Case presentation

A young woman in her 20s presented to the primary care physician's office with concerns about green discoloration in her right index fingernail. She reported no associated pain in her finger, but on physical examination, it presented with mild erythema and inflammation. The patient had recently undergone treatment for otitis media, otitis externa, and parotitis due to *P. aeruginosa *about two weeks ago in the emergency department.

The patient's recent medical history revealed that she first visited the urgent care to seek medical attention for a sore throat and a swollen left tonsil a couple of days prior to the emergency room (ER) visit. She was prescribed amoxicillin for her throat infection for seven days. However, she began to experience ear pain the next night, which worsened throughout the day. Subsequently, she sought emergency care at the hospital due to severe ear pain.

**Figure 1:**
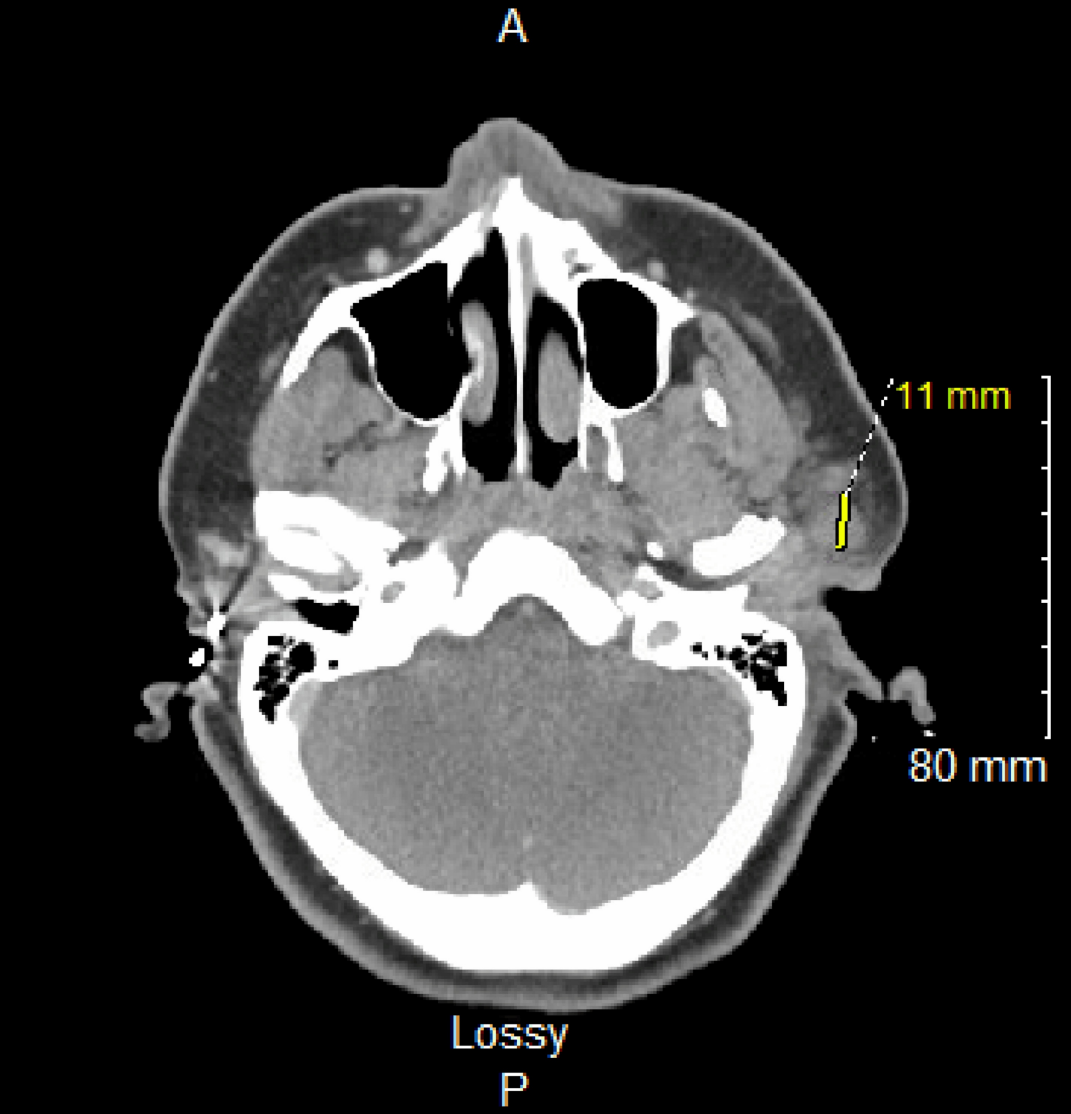
The head and neck CT scan indicated soft tissue density within the left external and middle ear consistent with otitis media and externa with parotid involvement.

On initial presentation, the patient was tachycardic with no respiratory distress. The physical exam revealed left ear swelling, tenderness, and cervical lymphadenopathy. The rest of the physical exam was unremarkable. Laboratory investigations revealed hyperkalemia, leukocytosis, and elevated alanine aminotransferase (ALT). The culture of the affected ear effusion confirmed *P. aeruginosa* with sensitivity to ciprofloxacin, gentamicin, and tobramycin. The head and neck CT scan showed soft tissue swelling due to inflammation, which confirmed the diagnosis of otitis media and externa with parotid involvement (Figure 1). The clinical evaluation revealed left-sided parotitis associated with ipsilateral acute otitis media and otitis externa, all attributed to *P. aeruginosa* infection.

She was discharged with a 10-day treatment plan comprising ciprofloxacin (500 mg twice daily) for targeted antibiotic coverage against *P. aeruginosa*, clindamycin (300 mg every eight hours) for broader coverage including anaerobic bacteria commonly found in ear and parotid infections, morphine for pain management, and ondansetron for nausea control.

**Figure 2:**
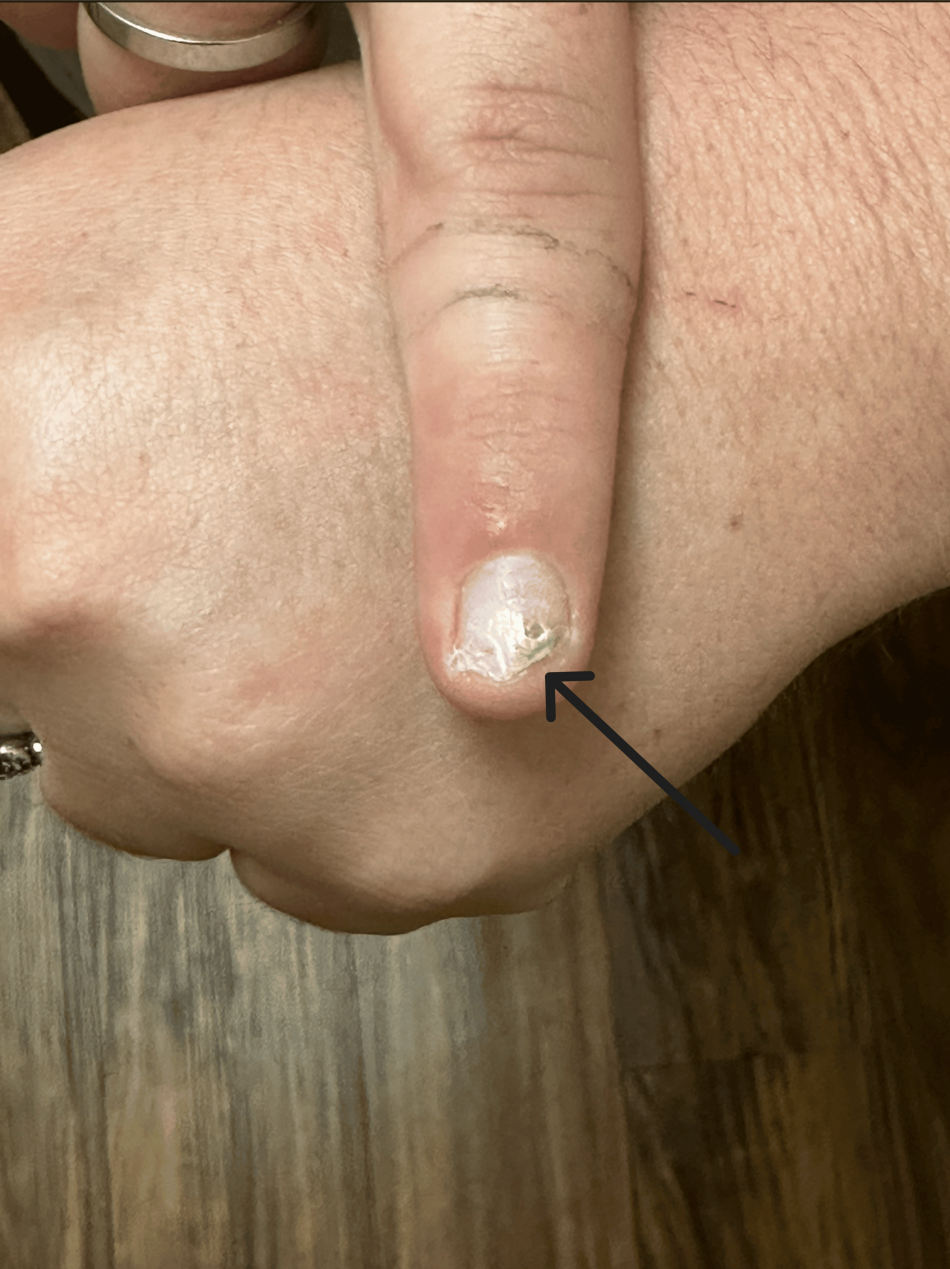
Infected nail bed caused by Pseudomonas aeruginosa.

On a subsequent visit, she mentioned the development of a green nail on her right index finger, which was suspected to be related to her recent ear infection (Figure 2). Upon examination, the patient's right second fingernail exhibited a prominent green discoloration on the distal aspect, more pronounced on the radial side. There were no signs of significant subungual debris, but mild inflammatory changes were noted. An indentation was noted on the nail plate adjacent to the green discoloration, which was not full thickness. The nail plate was non-tender and firmly attached to the underlying nail bed.

As the nail was still intact and there was a lack of any significant inflammation, we did not culture for *P. aeruginosa*. However, based on the patient's recent history of confirmed *P. aeruginosa* ear infection and the characteristic green discoloration of the nail, *P. aeruginosa* infection was determined to be the most probable cause of the patient's chloronychia.

## Discussion

Green nail syndrome has the characteristic greenish color of the nail from the pigments pyoverdine and pyocyanin produced by *Pseudomonas* [3]. *P. aeruginosa* is an opportunistic gram-negative aerobic coccobacillus. It colonizes naturally damp areas such as soil, water, plants, and animals (including humans) [3-5]. In the current patient, *P. aeruginosa* was confirmed in the ear culture as shown in Table 1.

**Table 1:** Ear culture results.

Organism	Growth	Antibiotic susceptibility
Pseudomonas aeruginosa	Heavy	Sensitive: ciprofloxacin, gentamicin, tobramycin
		Resistant: amoxicillin, amoxicillin-clavulanate

Due to local trauma, *P. aeruginosa* takes advantage of defective barriers, such as broken skin or nail beds [6]. The patient's green nail syndrome most likely occurred when the bacterium spread from her ear to her right index finger, potentially by unintended contact or shared fomites [5]. Hence, we suspect the origin was the otitis media/externa and parotitis.

Green nail syndrome is most commonly observed in immunocompromised adults or in patients who have predisposing factors, including, but not limited to, microtrauma, trichotillomania, and nail disorders like psoriasis [3,7]. Epidemiological studies indicate that only 5-8% of cases occur in individuals under the age of 30 years [3]. Our patient had no past medical history and a normal body mass index, regularly exercised, and followed a low-fat, high-protein diet.

Green nail syndrome is more common in certain work environments, such as barbers, dishwashers, bakers, and medical staff, where workers consistently come into contact with water, and it is considered an occupational disease [3]. The prevalence rate among healthcare workers has been reported to be 2-5 times higher than in the general population [7].

If left untreated, *P. aeruginosa* infection can cause complications such as malignant external otitis, endophthalmitis, endocarditis, meningitis, pneumonia, and septicemia. The likelihood of recovery from such infections is directly related to the severity of the underlying disease process. When left untreated, there is a high incidence of recurrence, spread, or damage/removal of the nail bed, resulting in permanent nail deformities [6].

Treatment of green nail syndrome or chloronychia consists of a multifaceted approach. Commonly, topical gentamicin solution has been an effective, inexpensive treatment for this condition. The challenge of treatment stems from the bacteria's production of biofilm [8]. When a single antibiotic is ineffective, therapeutic efficacy has been improved from the combination of gentamicin/ciprofloxacin and tobramycin/clarithromycin [7].

Our approach with this patient was conservative due to her recently completing an antibiotic course and the mild severity of her symptoms. The recommendations were topical ciprofloxacin (twice daily application for two weeks) and water/bleach soak (1:4 ratio of household bleach to water for 15 minutes twice daily) of the affected finger. The patient was followed up after three weeks and showed complete resolution of the green discoloration and associated inflammation.

Chloronychia due to *P. aeruginosa* can be recognized easily by simple clinical observation of the color of the nails to avoid unnecessary laboratory investigations, hence saving cost to the patient and time. However, in cases with atypical presentation or treatment failure, nail culture and antimicrobial susceptibility testing should be considered.

## Conclusions

Chloronychia, often known as green nail disease, is a rare yet clinically intriguing disorder caused by *P. aeruginosa*. The bacterium's opportunistic character is highlighted in this case by its presence in a younger patient with a recent history of *Pseudomonas *ear infections. While chloronychia is often associated with chronic paronychia in older individuals or those with occupational exposure, its emergence in a younger patient with a history of* P. aeruginosa* ear infections highlights the importance of comprehensive examination and tailored treatment techniques.

Early identification, timely treatment, and addressing underlying risk factors are critical to preventing complications and promoting recovery. Close monitoring and patient education on preventive actions are critical to achieving curative results. This case underscores the need for clinicians to consider the possibility of cross-site infection with opportunistic pathogens like *P. aeruginosa*.
